# Dietary diversity and micronutrients adequacy among the women of reproductive age at St. Martin’s island in Bangladesh

**DOI:** 10.1186/s40795-023-00715-y

**Published:** 2023-03-21

**Authors:** Md. Hafizul Islam, Ahmed Jubayer, Abira Nowar, Md. Moniruzzaman Nayan, Saiful Islam

**Affiliations:** 1 Institute of Nutrition and Food Science, University of Dhaka, Dhaka, 1000 Bangladesh; 2 Bangladesh Institute of Social Research (BISR) Trust, Dhaka, 1207 Bangladesh

**Keywords:** Dietary diversity, Micronutrients intake, Women of reproductive ages, St. Martin’s island, Bangladesh

## Abstract

Minimum dietary diversity for women, an important dimension of diet quality, has been widely used as a proxy indicator for micronutrient adequacy. In low- and middle-income countries (LMICs), women of reproductive age (WRA) particularly are at high risk of inadequate micronutrient intake resulting from poor diversified diets. Therefore, the present study aimed to assess dietary diversity and micronutrients adequacy in the diets of WRA of St. Martin’s island, along with their socio-economic determinants. A cross-sectional study was conducted on a representative sample of 201 WRA living at St. Martin’s island. Utilizing the Estimated Average Requirement (EAR) cut-point approach, the adequacy of micronutrient intake was evaluated from observed 24-h recall dietary data. The recent guideline of FAO was employed to evaluate Minimum Dietary Diversity for Women (MDD-W). Binary logistic regression and multiple linear regression analyses were performed to identify socio-economic determinants of MDD-W and micronutrients adequacy. The mean (SD) dietary diversity score was 4.25 (1.17) and about 40.3% of the participants met the MDD-W cut-off. Starchy staples (100%), meat/poultry/fish (87%), and other vegetables (79%) were consumed more frequently, while, the least reported food groups were dairy (2%), nuts and seeds (11%), and vitamin A-rich fruits and vegetables (11%). Except for Niacin, intake of all micronutrients was inadequate, with an inadequacy prevalence of 36–100%. Educational level, and decision-making role of women were significantly related to their dietary diversity. On the other hand, age, decision-making role, and MDD-W were important determinants of micronutrient adequacy. In conclusion, the WRA of St. Martin's island consumed neither a sufficient amount of micronutrients nor an adequate diversity of foods. In addition, several socio-economic components are linked with dietary diversity and micronutrient adequacy. Therefore, attention is needed to decide on the best strategies to improve the quality of diet and dietary diversity for WRA in this setting.

## Introduction

Maternal and child malnutrition is one of the major public health concerns in Bangladesh with a high prevalence of maternal micronutrients deficiency, in particular anemia and vitamin A deficiency [1–3]. Women of reproductive age (15–49 years) require proper nutrition and health status both for their own and their offspring through safe pregnancy, safe delivery, and better pregnancy outcomes [4]. They require additional nutrition during pregnancy and lactating periods to support extra physiological requirements [5]. However, along with genetic and environmental issues, the nutritional and health status of reproductive aged women relies on their dietary practices [6]. High-quality and optimal diversified diets are thought to be the ideal vehicle for ensuring an adequate supply of micronutrients [7, 8]. Insufficient dietary intake both in terms of quality and quantity before and during pregnancy and lactation can affect both women and their infants. Diversified diets and adequate dietary micronutrients intake, therefore, are to be prioritized for this crucial age group.

Poor dietary intake, and less diversified foods during pregnancy contribute to maternal malnutrition in low and middle-income countries (LMICs) [9, 10]. Deficiencies of various micronutrients especially Iron, Vitamin A, Folate, Calcium, and Zinc aggravate the conditions by many folds. Evidence strongly suggests that when maternal malnutrition exists, the offspring are at risk of low birth weight, decreased neonatal linear growth, stunting, long-term impaired cognition, delayed mental development, and neonatal death [11]. Therefore, they are to enter pregnancy with optimal health status and micronutrients storage.

Therefore, micronutrients fortification programs, and home gardening programs have been launched in different LMICs, including in Bangladesh, to improve the micronutrients status of the vulnerable [12]. National Micronutrient survey of Bangladesh (NMS-2011–2012) and other studies have reported inadequacy of several micronutrients intake and biochemical deficiencies of Vitamin A, Iron, and Zinc among reproductive-aged women of rural Bangladesh [13–15]. Recent evidence in Bangladesh shows that 69% of WRA consume inadequately diverse diets (less than four food groups out of nine or ten) [15, 16]. Micronutrients intake, therefore, tends to be inadequate among the majority of women of reproductive age in Bangladesh. Recent studies reflect that majority of WRA (adolescents: 73.1–88.5%; adult women: 72.9–87.6%) in Bangladesh had an inadequate intake of Vitamin A, Folic acid, Vitamin B_12_, and Calcium [17, 18]. Micronutrients deficiency disorders, therefore, are likely to be prevailing among WRA in Bangladesh [14]. Since women in resource-poor settings, often consume poor-quality, and cereal-based monotonous diets, they are thought to be at high risk for inadequate micronutrient status [19]. Inadequate dietary diversity along with other socio-economic factors explain low micronutrients adequacy in rural Bangladesh [8, 17].

St. Martin’s island, the most south-easterly spot of Bangladesh, the only coral-bearing tropical island of the country, is one of the most popular destinations for tourists all over the world due to its natural beauty, ecological, and biodiversity value. Most of the inhabitants living here lead their lives primarily by fishing and tourism business [20]. All year round they have to maintain communications with the mainland (Teknaf, Cox’s Bazar) via a sole transportation system, trawler/water boat, for daily purposes and they are fully marooned in this island during the monsoon. Moreover, they have to depend mainly on imported foods from the mainland rather than their own production which makes it challenging for them to have optimal diversified and nutritionally adequate diets. Therefore, they have distinct lifestyles and livelihoods separated from those of mainland of Bangladesh. Despite the enormous human settlement, it is unfortunate that very little is known about their health, nutrition, and dietary practices since this community hasn’t been brought under any health or nutrition survey. However, recent studies explored household water, sanitation, hygiene (WASH) condition, acute diarrhea, and the prevalence of malnutrition among children under 5 years of age. They highlighted 36.5% of the household without better sanitation, and about 1 in 4 children under 5 years of age (26.4%) having acute diarrhea [21, 22]. They also reported child malnutrition in St. Martin’s island with a high prevalence of stunting (34.4%), wasting (17.6%), underweight (18.9%) and 6.9% of the children as overweight [21, 23]. Along with some other factors, poor dietary practices of the women lead to maternal malnutrition which could contribute to child malnutrition (low birth weight, stunting, wasting, underweight, cognitive impairment etc.) [11].

To our knowledge, there is no study published in the literature which includes the dietary diversity and intake of micronutrients among the reproductive-aged women of this island. Therefore, the present study focused on the dietary diversity of reproductive-aged women, their dietary micronutrients intake, and adequacy as compared to the recommended intake. Furthermore, the socio-economic determinants of the minimum dietary diversity, as well as the adequacy of micronutrients, were also investigated. It is anticipated that this study will fill the research gap by providing information on dietary diversity and dietary micronutrients intake of WRA residing in St. Martin’s island. This will in turn assist in the implementation of maternal nutrition and reproductive health program to improve their health conditions and dietary nutrient intake.

## Methodology

### Study location and subjects

A community-based cross-sectional survey was conducted from late February to early March, 2020 among the residents of St. Martin’s island residing in non-institutional dwelling units on the island. The study covered all the nine wards/villages of St. Martin’s island namely Deil Para, Dakshin Para, Zinzira, Konar Para, Majher Para, Nazrul Para, Purba Para, Paschim Para, and Uttar Para.

### Sample design and sampling procedure

There were nine administrative villages or wards with a total of 1169 households at St. Martin’s island. This study was a part of nutrition study among WRA of this island and a representative sample of 201 women were included in the study. The sample size was calculated based on the prevalence of underweight (13.2%) among rural women of Bangladesh using Cochran’s formula at a 95% confidence interval and a 5% degree of precision. The study was based on a stratified sample of households; each village was designated as a stratum. With proportionate allocation, the entire sample was distributed into nine strata (nine strata because there are nine villages). A complete list of the households was unavailable which restrained us from using simple random sampling. Instead, a modified Expanded Program of Immunization (EPI) sampling approach was applied to select the surveyed households [24]. Only one WRA was chosen at random from a family with more than one WRA.

### Dietary data collection and nutrient intake analysis

The enumerators were nutrition graduates with experience in data collection for nutrition surveys. Demographic details and detailed dietary information were recorded using the printed form of a pre-tested, validated questionnaire. Dietary data were collected by a single 24-h recall method at the household level, a validated approach for obtaining necessary dietary information to calculate nutrient intake and adequacies [25, 26]. Respondents were asked about all food items consumed by themselves either at their household or outside of the home in the previous 24 h. They were further asked to provide the approximate cooked or raw weight of each food item or dish.

The Bangladeshi Food Composition Table (FCT) [27, 28] was used to calculate intakes of energy and micronutrients (Vitamin A, Thiamin, Riboflavin, Niacin, Vitamin B_6_, Vitamin B_12_, Folate, Vitamin C, Iron, Calcium, and Zinc). Nutrient content of those foods (e.g. Salmon fish), which were not available in the local FCT, was adopted from the Indian Food Composition Table (IFCT) [29]. Vitamin B_12_ contents of foods were obtained from the USDA food database (*Food Data Central*) and another source [31].

As food acquisition data were obtained at the household level, individual intake was measured using the Adult Male Equivalent (AME) approach. Appropriate AMEs were assigned to each household member, weighed according to their age and sex [32]. Then, per capita nutrient consumption was calculated using Eq. 1. Although this AME approach has certain limitations, it is an invaluable way to make inferences on individual nutrient intakes and adequacies from household-based food consumption surveys [33].

1 $$\mathrm{Per\,capita\,nutrient\,consumption}= \frac{Total\,household\,consumption}{Total\,consumption\,unit} \times corresponding\,man\,value$$

### Assessment of micronutrient adequacy

To calculate micronutrients adequacy in the diet of WRA, the dietary micronutrient intake of the WRA was compared with the age- and sex-specific Estimated Average Requirement (EAR), recommendations of the Indian Council of Medical Research (ICMR) [34]. In addition, Nutrient Adequacy Ratio (NAR) was calculated for each nutrient which is the ratio of an individual’s intake to the age- and sex-specific recommendations (Eq. 2) [35]. Following previous studies [17, 36], the Mean Adequacy Ratio (MAR) was calculated using these NAR values as an overall measure of nutrient adequacy. NAR values were truncated at 1 to prevent masking of the deficiency of another nutrient with NAR far below 1. When NAR was equal to or above 1, it was truncated to 1. When NAR was below 1, it was truncated to zero (0). The MAR was then calculated by averaging all the truncated NAR values together (Eq. 3). Thus, the MAR was reported on a scale from 0 to 1, with 0 indicating that the requirement for no nutrients was met, and 1 indicating that the requirements for all nutrients were met.

2 $${\varvec{N}}{\varvec{A}}{\varvec{R}}=\frac{Daily\,dietary\,nutrient\,intake}{Estimated\,average\,requirement\,of\,that\,nutrient}$$

3 $${\varvec{M}}{\varvec{A}}{\varvec{R}}=\boldsymbol{ }\frac{\sum NAR (truncated\,to\,1\,or\,0)}{Number\,of\,nutrients}$$

### Assessment of dietary diversity

The Minimum Dietary Diversity for Women of reproductive age (MDD-W) guideline was used for collecting data on food intake over the previous 24 h [37]. A list of 10-food groups as proposed by the MDD-W guideline consists of *1*) Starchy staples, *2*) Beans and peas, *3*) Nuts and seeds, *4*) Dairy products (milk, yogurt, and cheese), *5*) Flesh foods (meat, fish, poultry, and liver or organ meats), *6*) Eggs, *7*) Dark green leafy vegetables, *8*) Vitamin A-rich fruits and vegetables, *9*) Other vegetables and *10*) Other fruits. The respondents were asked whether they consumed the foods from this list during the previous 24 h. Foods whose total consumption during the previous day was ≥ 15 g were considered and included in the food groupings.

Each group was assigned a score of 1 if it had been consumed, otherwise, coded as 0. The ten food groups were first summed into a score ranging from 0 to 10. According to the FAO guideline, those who consumed at least 5 different food groups were considered to meet adequate dietary diversity [37].

### Statistical analyses

The normal distribution of the variables was investigated before analysis using statistical test- Kolmogorov–Smirnov and Shapiro–Wilk test as well as visual inspection of the histogram, and Q-Q plot. Dietary intake data and most of the socio-demographic data were not normally distributed. Therefore, descriptive statistics for continuous variables have been presented as the median and Inter-quartile range (IQR), and frequency and percentages (%) have been used for categorical variables. A binary logistic regression model was used to explore the determinants of MDD-W. To identify the determinants of micronutrients intake adequacy, multiple linear regression analysis was conducted including all possible covariates. A p-value of less than 0.05 was considered to be statistically significant. Data were analyzed using the Statistical Packages for Social Sciences, SPSS (Version 25.0).

### Study variables

The selection of potential socio-demographic explanatory variables was based on the literature which reported factors of dietary diversity and micronutrients intake adequacy of women [8, 16, 38, 39]. Dietary energy intake, age, marital status, BMI, and decision-making role of women were included as individual-level factors while household income quartiles, household size, and household food security were included as household-level factors. To assess household food insecurity, an experience-based Food Insecurity Experience Scale (FIES) was employed following the United Nations Food and Agriculture Organization (FAO) guideline [40]. Anthropometric information (height and weight) of WRA were collected using a portable height scale, and weight machine. Weight and height were taken to the nearest 0.1 kg, and 0.01 m respectively. The nutritional status of WRA was then determined based on the WHO Body Mass Index (BMI) cut-off; underweight: BMI < 18.5, normal: BMI 18.5–24.99 and overweight/obesity BMI ≥ 25. Women’s decision-making role was measured by the women’s participation in the following three decisions: determining own health care; making large household purchases; and visiting family or relatives. Score 1 was assigned if a participant had access to decision making otherwise scored 0. Thus, a scale of 0–3 was obtained for the variable, decision making role of women. Those who could participate in all these issues were considered to have decision-making role otherwise not. Finally, they were categorized as having a decision making role (score: 3) and not having a decision making role (score: 0–2).

### Regression model building

#### Binary logistic regression model

A binary logistic regression model was used to explore the determinants of MDD-W. Before constructing the final model, the fundamental assumption of the logistic regression model, namely multicollinearity and model stability, was thoroughly evaluated. Pearson goodness of fit was used to verify the regression model's validity [41]. The variance inflation factor (VIF) was used to check for multicollinearity in the model, with VIF larger than two indicating multicollinearity.

### Multiple linear regression model

An exploratory analysis to identify the determinants of micronutrients intake adequacy with multiple linear regression analysis was conducted including all possible covariates. The Mean adequacy ratio (MAR) was the outcome variable for the multiple linear regression analysis. Variance inflation factor (VIF) was examined to check the amount of multicollinearity in the model and VIF greater than two was considered to indicate multicollinearity. The residual for each case was calculated and the normality of residuals was inspected through the residual plot as well as other normality check procedures, such as descriptive statistics, normality plot, and statistical test. Cook’s distance and leverage values were observed to identify multivariate outliers. The critical value for Cook’s distance was set to 1 which is 0.05 for leverage value.

### Ethical approval

The nature and purpose of our study was explained to all the study participants in detail and oral inform consent was taken before the interview. Since most of the respondents were illiterate, written consent couldn’t be taken from them and oral inform consent is approved by the ethical review committee of the Faculty of Biological Sciences, University of Dhaka (Ref. No. 116/Biol. Sci.). For illiterate participants and participants whose age is below 16 years old, informed consent was obtained from their literate legal guardians.

## Results

### Socio-economic characteristics

The socio-economic characteristics of the respondents have been presented in Table 1. The mean (SD) age of the participants was 34.7 (8.59) years and more than half (52.7%) of them were above 35 years. Among the villages, higher percentages of women were from *Dakshin para* (16%) followed by *Konar para* (15%), and *Purba para* (13%). About 85% of the study participants were married. Most of them had inadequate formal education (36.8% never attended school) and only 7.5% reached secondary education. The majority of them were housewives (94%) and were found to have a decision-making role in important family affairs (63.2%). About 44% of the households had more than 5 members. About 56% of the households were food secure. The prevalence of underweight was low (7%) while the rate of overweight/obesity was higher (38%).

**Table 1:** Socio-economic characteristics of the study subjects (*n* = 201)

Characteristics	n (%)
**Age (years)**	
Mean (SD)	34.7 (8.59)
15–24	23 (11.4)
25–34	72 (35.8)
35–49	106 (52.7)
**Place of residence**	
Purba para	26 (12.9)
Deil para	24 (11.9)
Uttar para	19 (9.5)
Paschim para	17 (8.5)
Zinzira	7 (3.5)
Konar para	29 (14.4)
Nazrul para	19 (9.5)
Dakshin para	35 (17.4)
Majher para	25 (12.4)
**Marital status**	
Married	170 (84.6)
Unmarried	31 (15.4)
**Educational level**	
No formal education	74 (36.8)
Up to primary	112 (55.7)
Up to secondary	15 (7.5)
**Occupation**	
Housewife	189 (94.0)
Others	12 (6.0)
**Household income quartiles**	
Lowest	52 (25.9)
Second	63 (31.3)
Third	45 (22.4)
Highest	41 (20.4)
**Household size**	
≤ 5 people	112 (55.7)
> 5 people	89 (44.3)
**Women’s decision-making role**	
Yes	127 (63.2)
No	74 (36.8)
Nutritional Status*	
Underweight	14 (7.0)
Normal	110 (54.7)
Overweight	77 (38.3)
**Household food security**	
Food Secure	112 (55.7)
Food Insecure	89 (44.3)

Underweight: BMI < 18.5, Normal: BMI 18.5–24.99 and Overweight/obesity BMI ≥ 25.0

^*^Nutritional status was determined based on the WHO BMI cut-off

### Dietary diversity characteristics

The percentage of WRA consuming different food groups has been listed in Table 2. The mean (SD) dietary diversity score (DDS) was 4.25 (1.17) and only 40.3% of the study subjects were found to consume adequate diversified foods (at least 5 different food groups). Starchy staples, meat/fish/egg, and other vegetables were most commonly consumed, while, dairy products, eggs, vitamin A-enriched fruits, and vegetables were reported by few individuals. Cereals based foods were consumed by all the women (100% in both groups) irrespective of their dietary diversity. Percentages of women consuming all types of food groups, except dairy products, and meat/fish/poultry, were significantly higher among adequate diversified groups compared to the inadequate.

**Table 2:** Percentage of WRA consuming foods from various food groups (*N* = 201)

	**Total** **(*n* = 201)**	**Inadequate dietary diversity (*n* = 120)**	**Adequate dietary diversity (*n* = 81)**	*p*-value*
Proportion consumed ≥ 5 food groups	40.3	-	-	
Starchy staples	100	100	100	-
Pulses	35.3	18.5	59.8	< 0.001
Nuts and seeds	10.9	5.0	19.5	0.002
Dairy product	2.0	1.7	2.4	1.00
Meat/poultry/fish	86.6	83.2	91.5	0.098
Egg	21.9	10.9	37.8	< 0.001
Dark green leafy vegetables	35.8	21.0	57.3	< 0.001
Vitamin A-rich fruits and vegetables	10.9	5.0	19.5	0.002
Other vegetables	78.6	70.6	90.2	0.001
Other fruits	43.3	28.6	64.6	< 0.001

^*^p-value from chi-square test

### Determinants of minimum dietary diversity of WRA

Socio-economic determinants of minimum dietary diversity of the women have been presented in Table 3. The educational level, and their decision-making role in important family affairs were significantly related to their dietary diversity. The odds of women having adequate diversified diets were higher among those who had at least secondary education compared to those who never attended school (AOR = 7.20, 95% CI = 1.84-28.15, *p* = 0.005). Moreover, women having decision-making roles in the family had more chance (AOR = 2.44, 95% CI = 1.19-5.03, *p* = 0.015) to take diversified foods compared to others.

**Table 3:** Logistic regression predicting the Minimum Dietary Diversity of Women (MDD-W)

Variables	Minimum dietary diversity		AOR (95% CI)*	*P*-value
	Adequate n (%)	Inadequate n (%)		
**Age (years)**				
15–24	8 (34.8)	15 (65.2)	1	
25–34	35 (48.6)	37 (51.4)	2.05 (0.67, 6.30)	0.210
35–49	38 (35.8)	68 (64.2)	0.95 (0.24, 3.08)	0.930
**Village**				
Zinzira	2 (25.0)	6 (75.0)	0.67 (0.09, 5.02)	0.693
Dakshin para	14 (41.2)	20 (58.8)	1.78 (0.47, 6.73)	0.379
Uttar para	6 (31.6)	13 (68.4)	0.70 (0.15, 3.19)	0.645
Purbo para	12 (46.2)	14 (53.8)	1.37 (0.35, 5.27)	0.651
Majher para	10 (40.0)	15 (60.0)	1.77 (0.41, 7.55)	0.44
Deil para	7 (31.4)	16 (69.6)	0.76 (0.17, 3.39)	0.715
Paschim para	10 (58.8)	7 (41.2)	3.44 (0.74, 16.10)	0.117
Konar para	14 (48.3)	15 (51.7)	2.08 (0.53, 8.18)	0.295
Nazrul para	6 (30.0)	14 (70.0)	1	
**Marital status**				
Married	71 (41.8)	99 (58.2)	1.23 (0.47, 3.26)	0.677
Unmarried	10 (32.3)	21 (67.7)	1	
**Educational level**				
No formal education	24 (32.4)	50 (67.6)	1	
Up to primary	46 (41.1)	66 (58.9)	1.72 (0.82, 3.58)	0.149
Up to secondary	11 (73.3)	4 (26.7)	7.20 (1.84, 28.15)	0.005
**Occupation**				
Housewife	75 (39.7)	114 (60.3)	1	
Others	6 (50.0)	6 (50.0)	2.14 (0.59, 6.87)	0.211
**Income quartiles**				
Lowest	21 (40.4)	31 (59.6)	1	
Second	21 (33.3)	42 (66.7)	0.77 (0.33, 1.79)	0.537
Third	18 (40.0)	27 (60.0)	1.38 (0.54, 3.53)	0.504
Highest	21 (51.2)	20 (48.8)	1.81 (0.65, 5.01)	0.255
**Household size**				
≤ 5 people	45 (40.2)	67 (59.8)	0.98 (0.50, 1.96)	0.964
> 5 people	36 (40.4)	53 (59.6)	1	
**Women’s decision making role**				
Yes	56 (44.1)	71 (55.9)	2.44 (1.19, 5.03)	0.015
No	25 (33.8)	49 (66.2)	1	
**Nutritional status**				
Underweight	3 (21.4)	11 (78.6)	0.53 (0.11, 2.43)	0.329
Normal	44 (40.0)	66 (60.0)	1	
Overweight	34 (44.2)	43 (55.8)	1.37 (0.65, 2.79)	0.472
**Food Security Status**				
Food Secure	44 (39.3)	68 (60.7)	0.81 (0.38, 1.71)	0.576
Food Insecure	37 (41.6)	52 (58.4)	1	

^*^AOR Adjusted odds ratio, *CI* Confidence interval, *MDD-W* Minimum dietary diversity for women; Reference for dependent variable: inadequate dietary diversity; Significant at *p*-value < 0.05

### Dietary micronutrients intake and adequacy as compared to recommendation

Dietary intake of various micronutrients and their adequacy as compared to the Estimated Average Requirement (EAR) have been demonstrated in Table 4. The median intake of Vitamin A, Vitamin C, Riboflavin, Folate, Iron, and Calcium was inadequate compared to their dietary recommendations (EAR). None of the respondents met the EAR of Riboflavin and a high percentage of the respondents consumed inadequate amount of Vitamin A (84.6%), Vitamin C (66%), Thiamin (50%), Folate (91%), Calcium (83.6%), Iron (85%), and Zinc (72%). Only Niacin intake was satisfactory meeting adequacy for almost all of the WRA. Vitamin A, and Calcium intake covered only 21% and 36% respectively of their dietary recommendation. The mean (SD) adequacy ratio of 11 micronutrients was 0.43 (0.13).

**Table 4:** Dietary micronutrients intake and adequacy as compared to recommendation

Nutrients	EAR	TUL	Median (25^th^, 75^th^)	Percentage with Intake		Median NAR*	MAR* Mean (SD)
				Below EAR*	Above TUL*		
Vitamin A (µg/day)	390	3000	80.4 (29.5, 214.8)	84.6	0.5	0.21	0.43 (0.13)
Vitamin C (mg/day)	55	2000	45.2 (27.8, 63.9)	66.2	0.0	0.82	
Thiamin (mg/day)	1.4	NA	1.4 (1.2, 1.6)	50.2	-	0.99	
Riboflavin (mg/day)	2	NA	0.7 (0.6, 0.9)	100.0	-	0.37	
Niacin (mg/day)	12	35	34.4 (27.5, 39.8)	0.5	44.8	2.67	
Vitamin B_6_ (mg/day)	1.6	100	1.8 (1.5, 2.3)	35.8	0.0	1.15	
Folate (µg/day)	180	1000	116.9 (87.4, 145.1)	91.0	0.0	0.65	
Vitamin B_12_ (µg/day)	2.0	NA	2.9 (1.16, 5.5)	40.3	-	1.49	
Calcium (mg/day)	800	2000	283.5 (155.8, 645.0)	83.6	0.5	0.35	
Iron (mg/day)	15	45	9.5 (6.9, 12.9)	84.6	0.0	0.64	
Zinc (mg/day)	11	40	9.6 (8.3, 11.4)	71.6	0.0	0.87	

^*^EAR Estimated Average Requirement, *TUL* Tolerable upper level, *NAR* Net adequacy ratio, *MAR* Mean adequacy ratio, *SD* Standard deviation; 25^th^ percentiles and 75^th^ percentiles

### Major contributing food groups to different micronutrients

Table 5 illustrates the major contributing food groups to energy and different micronutrients intake. A major portion of total dietary energy (about 69%) and dietary B vitamins came from cereal-based products mainly rice and wheat. Almost 83% of Vitamin C and two-thirds of Vitamin A were covered from fruits and vegetables. Fruits and vegetables also contributed to dietary Folate (35%), Iron (19%), Riboflavin (17%), Calcium (16%) and Zinc (9%). A greater portion of Vitamin B_12_ (82%), Calcium (72%), Riboflavin (47%), and Iron (39%) came from animal sources (meat, fish, and egg). Dairy products mainly milk contributed to 18% of Vitamin B_12_.

**Table 5:** Major contributing food sources of energy and different micronutrients

Nutrients	Cereals	Nuts and legumes	Fruits and vegetables	Meat, fish and egg	Dairy
Energy	68.96	1.78	2.66	15.13	0.52
Vitamin A	-	0.50	66.50	33.00	-
Vitamin C	-	1.20	82.89	2.50	-
Thiamin	68.77	6.49	5.42	16.95	-
Riboflavin	30.65	2.54	17.06	46.63	0.20
Niacin	60.11	2.02	3.84	33.08	0.30
Vitamin B_6_	57.45	4.22	5.32	26.78	0.21
Folate	41.88	5.58	35.20	11.34	0.50
Vitamin B_12_	-	-	-	82.48	17.55
Calcium	9.82	0.83	15.64	72.41	1.98
Iron	32.49	7.07	19.19	38.96	0.20
Zinc	60.71	4.76	9.02	24.45	0.67

### Determinants of micronutrients adequacy of WRA

Socio-economic determinants of mean adequacy ratio (MAR) of micronutrients intake have been presented in Table 6. Both univariate and multiple linear regression models were used to find the major contributing determinants. MAR was positively associated with dietary energy intake, age, decision-making role and adequate dietary diversity. Micronutrient adequacy was significantly higher among women of 25–35 years compared to the young women. Minimum dietary diversity was also a good predictor of the adequacy of micronutrients.

**Table 6:** Determinants of micronutrients adequacy of WRA

	Univariate linear regression model		Multiple linear regression model	
	**Coefficient (95% CI)**	***p*-value**	**Coefficient (95% CI)**	***p*-value**
**Energy intake (Kcal)**	0.000^a^	< 0.001	0.000^b^	< 0.001
**Age (years)**				
15–24	Ref		Ref	
25–34	0.015 (-0.046, 0.075)	0.632	0.061 (0.007, 0.116)	0.027
35–49	0.029 (-0.011, 0.069)	0.153	-0.004 (-0.038, 0.030)	0.826
**Household size**				
Small (≤ 5 people)	-0.054 (-0.091, -0.017)	0.004	0.001 (-0.031, 0.034)	0.944
Large (> 5 people)	Ref		Ref	
**Women’s decision-making role**				
Yes	0.037 (0.011, 0.085)	0.060	0.030 (0.002, 0.061)	0.039
No	Ref		Ref	
**MDD-W**				
Inadequate (< 5 FG)	Ref		Ref	
Adequate (≥ 5 FG)	0.048 (0.011, 0.085)	0.012	0.049 (0.018, 0.079)	0.002
**Educational level**				
No formal education	Ref		Ref	
Up to primary	-0.003 (-0.043, 0.036)	0.871	-0.004 (-0.038, 0.029)	0.797
Up to secondary	0.037 (-0.037, 0.112)	0.328	-0.026 (-0.086, 0.035)	0.402
**BMI**				
Underweight	0.001 (-0.073,0.076)	0.971	0.038 (-0.023, 0.098)	0.225
Normal	Ref		Ref	
Overweight	0.034 (-0.005,0.073)	0.083	0.018 (-0.013, 0.049)	0.252

*CI* Confidence interval, *Ref* Reference

^a^Coefficient (95% CI): 0.000158 (0.000129, 0.000186)

^b^Coefficient (95% CI): 0.000163 (0.000133, 0.000194)

## Discussion

The study revealed the minimum dietary diversity and dietary micronutrients adequacy as compared to recommendations among the women of reproductive age in St. Martin’s island. The study also identified various socio-economic determinants of dietary diversity and dietary micronutrients adequacy. Our study findings indicated that both the mean dietary diversity score (4.25) of women and the proportion of women who reached the MDD-W (40.3%) were low. Women’s diet was largely dominated by starchy staples followed by meat/fish/egg and other vegetables, while, dairy products, eggs, vitamin A enriched fruits, and vegetables were reported by few individuals. Educational level, and decision-making role were significantly linked with their dietary diversity. Except for Niacin, all micronutrients were inadequate as compared to their EARs. Such inadequacies were reflected in the NARs and the MAR being low. Adequate dietary diversity, dietary energy intake, age, and the decision-making role of women were positively associated with micronutrients adequacy and were good predictors of it.

Although several studies reported MDD-W of Bangladeshi women, no studies prior to this one were found to include the dietary diversity of women from this island. The findings of the study indicate consensus with other studies focusing on MDD-W of Bangladeshi women which reported their low dietary diversity score (3.87 to 4.84) and a high percentage in the inadequacy of diversity (69%) [8, 16, 42]. Inadequately diversified diets among women of neighboring countries of Bangladesh, India (4.28 FG) [43], and Myanmar (4.09 FG) [44], are also reported in the literature. Women of reproductive age from other LMICs also consume inadequately diverse diets (3.0–3.82 FG) [45–52]. On the contrary, a high percentage of WRA (87%) of this island consumed flesh foods (mainly fish) than other Bangladeshi women [8, 17, 18, 53]. The reason behind this discrepancy could be explained by the availability of fish and seafood here and leading most of the households by fishing. Low intake of other foods especially dairy products, nuts and seeds, and vitamin A-rich fruits and vegetables was probably due to low production and less supply to this isolated area.

The educational level of WRA, and their decision-making role in important family affairs were significantly related to their dietary diversity. The odds of having adequate diversified diets were higher among those who had at least secondary education compared to those who never attended school. Moreover, women having decision-making roles in the family had more chances to take diversified foods compared to others. Similar studies among WRA of Bangladesh also highlighted that women’s schooling, and voice with husbands had a positive association with their dietary diversity, and households with vegetable gardens, rich households, and literate women were more likely to have better dietary diversity scores [16, 54].

The adequacy of dietary micronutrients intake of women as compared to their Estimated Average Requirements (EAR) was poor (MAR = 0.43). Several studies among the WRA in other parts of Bangladesh reported inadequate intake of dietary micronutrients [8, 17, 18]. Our study has some similarities in data collection methods, assessment tools for dietary diversity, dietary intake, and nutrient adequacy with another study in reproductive-aged adolescent girls and women in rural areas of Bangladesh [17]. The findings of the present study are consistent with the findings of that study which reported low MAR (0.49–0.51) in reproductive-aged adolescent girls and women in rural areas of Bangladesh [17]. These studies also reported low intakes of Vitamin A, Vitamin C, Riboflavin, Folate, Calcium, and Iron among them [8, 17, 18]. This indicates that dietary micronutrients intake adequacy among women is consistently low in Bangladesh. The findings from the present study support the fact that monotonous and plant-based diets which contain relatively lower quantities of micronutrients, contribute to the low adequacy of micronutrients [8, 55]. Women also tended to consume fewer dairy products and eggs which are one of the good sources of micronutrients. Moreover, low consumption of micronutrient-rich dark green leafy vegetables (by 36% WRA) contributed to low dietary inadequacy of most micronutrients. Very low consumption of milk and milk products by women also contributed to the low adequacy of their Riboflavin and Calcium intakes. Consistent with other studies dietary Vitamin A intake was very poor, 85% below the EAR, due to low consumption of vitamin A-rich fruits and vegetables in terms of both quantity and percentages of women [42, 55]. Dietary Riboflavin intake by none of the women of this island could reach the dietary recommendation due to low consumption of riboflavin-rich leafy vegetables, organ meat, and fruits. During the study period, the most available and mostly consumed fruits on this island were coconut and watermelon which are low in micronutrients contents. Vitamin C-rich citrus fruit intake tended to be consumed low that contributed to a lower intake of Vitamin C.

Micronutrient adequacy was significantly better with the increment of their age. Similar results were found in another study on Bangladeshi women and they reported that dietary quality was significantly better in adult women than in adolescent girls [17]. MDD-W was a good predictor of micronutrients adequacy and high dietary diversity signifies higher micronutrients adequacy which advocates the fact of considering dietary diversity as a proxy indicator of micronutrient adequacy. This result is consistent with other studies in Bangladesh and Burkina Faso [8, 52]. Moreover, micronutrients adequacy was significantly higher among women with a decision-making role in important family affairs. This finding support their more chances to consume diversified diets as discussed earlier. Moreover, women with decision-making role might have role in daily meal planning, expenditure on food, and meal distribution which contribute to both dietary diversity, and micronutrients adequacy.

Recent studies on children under 5 years of age of St. Martin’s island found a higher prevalence of stunting (34.4%), wasting (17.6%), and underweight (18.9%) among them [21, 23]. They also highlighted that less than half of them (45.5%) consumed an adequately diverse diet [21]. As mentioned earlier, poor dietary practices of the women along with other factors lead to maternal malnutrition which can contribute to child malnutrition [11]. This relationship is also supported by a higher prevalence of inadequate dietary diversity and multiple micronutrients inadequacy among women in our findings and higher prevalence of child malnutrition in other studies of St. Martin’s island. Thus, to improve the health condition of mothers and their children as well, the maternal diet should be underscored along with children’s. A more comprehensive study on mothers and child is recommended to draw a causal relationship between maternal dietary issues and child malnutrition.

### Strength and limitation

This is the first-ever study that explores dietary diversity and micronutrients intake among the women of reproductive age in the isolated Saint Martin's island of Bangladesh. However, the results should be considered in light of some limitations. Dietary data were collected using a single 24-h recall method which may not be the best estimation of usual intake but this is a validated approach for obtaining necessary dietary information to calculate nutrient intake and adequacies [25, 26]. Further studies should consider the risk of recall biases and use more sophisticated ways of assessing dietary intake, especially, when the population are mostly illiterate. Also, we did not collect individual dietary intake from the respondents and used the AME approach to find individual intake from household consumption. However, individual intake adopted from household food consumption through the AME approach reflects almost the same intake obtained from individual intake data [56]. Another study also advocates the AME approach to assess nutrient adequacy [57]. They concluded that the AME method could be a good proxy of individual dietary approach to calculate nutrient intake. However, we recommend further studies considering the shortcomings of the AME approach. In addition, in some cases, we had to depend on the external food composition table, especially the Indian Food Composition table but it might pose negligible variation in nutrients composition since geographical characteristics are almost the same for these two countries. Moreover, since it was a cross-sectional study, it provides a picture of a single moment of the island avoiding day-to-day or seasonal variation. However, cross-sectional studies may be a better source of data for policy judgments in the public health community than longitudinal studies when risk factors vary more across space at a fixed moment in time than at a fixed location across time [58]. Future studies should aim to capture seasonal and day-to-day variations in dietary consumption of women of reproductive age in this setting.

## Conclusion

Dietary diversity and micronutrients intake adequacy were poor among the WRA of Saint Martin’s island in Bangladesh. The least reported food groups were dairy products, nuts and seeds, and vitamin A enriched fruits and vegetables. Although Niacin, and vitamin B_6_ intake were satisfactory, intake of other micronutrients especially Vitamin A, Vitamin C, Riboflavin, and Calcium was very low as compared to their daily dietary recommendation. In addition, several socio-economic components are linked with dietary diversity and micronutrient adequacy. Hence, urgent attention is needed to decide on the best strategies to improve the quality of diet and dietary diversity for WRA in this setting. One potential approach could be community-based nutrition education intervention to educate and create awareness about the importance of diet for optimal health. Nutritional education interventions should emphasize the importance of consuming diversified diets and dark green leafy vegetables, vitamin A-rich fruits and vegetables, and dairy products along with fish/animal foods to ensure optimal micronutrients adequacy in this island.

## Data Availability

Original dataset cannot be shared right now since we have a plan to submit another research work using the same dataset. However, the data used for this current study are available from the corresponding author on reasonable request.

## Abbreviations

AME: Adult Male Equivalent
AOR: Adjusted Odds Ratio
BMI: Body Mass Index
CI: Confidence Interval
EAR: Estimated Average Requirement
EPI: Expanded Program of Immunization
FAO: United Nations Food and Agriculture Organization
FCT: Food Composition Table
FIES: Food Insecurity Experience Scale
ICMR: Indian Council of Medical Research
IQR: Inter-Quartile Range
LMICs: Low- and Middle-Income Countries
MAR: Mean Adequacy Ratio
MDD-W: Minimum Dietary Diversity for Women of Reproductive Age
NAR: Nutrient Adequacy Ratio
SD: Standard Deviation
SPSS: Statistical Package for Social Science
TUL: Tolerable Upper Level
VIF: Variance Inflation Factor
WHO: World Health Organization
WRA: Woman of Reproductive Age
